# P2Y_11_/IL-1 receptor crosstalk controls macrophage inflammation: a novel target for anti-inflammatory strategies?

**DOI:** 10.1007/s11302-023-09932-3

**Published:** 2023-04-05

**Authors:** Dominik Klaver, Martin Thurnher

**Affiliations:** Immunotherapy Unit, Department of Urology, Medical University of Innsbruck, Innrain 66a, 6020 Innsbruck, Austria

**Keywords:** Macrophage polarization, Inflammation, Homeostasis, NanoString

## Abstract

Although first cloning of the human ATP receptor P2Y_11_ was successful 25 years ago, the exact downstream signaling pathways of P2Y_11_ receptor, which can couple to G_q_ and G_s_ proteins, have remained unclear. Especially the lack of rodent models as well as the limited availability of antibodies and pharmacological tools have hampered examination of P2Y_11_ expression and function. Many meaningful observations related to P2Y_11_ have been made in primary immune cells, indicating that P2Y_11_ receptors are important regulators of inflammation and cell migration, also by controlling mitochondrial activity. Our recent studies have shown that P2Y_11_ is upregulated during macrophage development and activates signaling through IL-1 receptor, which is well known for its ability to direct inflammatory and migratory processes. This review summarizes the results of the first transcriptomic and secretomic analyses of both, ectopic and native P2Y_11_ receptors, and discusses how P2Y_11_ crosstalk with the IL-1 receptor may govern anti-inflammatory and pro-angiogenic processes in human M2 macrophages.

## Introduction

Purinergic signalling is initiated by adenosine-activated P1 receptors (A_1_, A_2A_, A_2B_, A_3_) and nucleotide-activated P2 receptors [1]. P2 receptors form two groups, the metabotropic G protein-coupled P2Y receptors (P2YRs) and the ionotropic P2X receptors (P2X_1-7_) [2]. Both, P1 receptors and P2YRs are G protein-coupled receptors (GPCRs) [3]. The huge family of GPCRs comprising more than 800 members can be grouped into 6 classes (A-F) depending on sequence homology and functional similarity [4]. The rhodopsin-like GPCRs constituting class A are not only by far the largest but also the best-characterized group. The rhodopsin/class A family can be subdivided into four main groups (α, β, γ, and δ). P2YRs belong to the δ group of the rhodopsin/class A family of GPCRs. From the eight known P2YRs, five „P2Y_1_-like “ P2YRs couple to G_q_ proteins (P2Y_1_, P2Y_2_, P2Y_4_, P2Y_6_, P2Y_11_,) and three „P2Y_12_-like “ receptors couple to G_i_ proteins (P2Y_12_, P2Y_13_, P2Y_14_). Like other cell surface receptors, P2YRs enable cells to continuously sense the extracellular milieu and to translate signals from outside indicating for instance stress and danger into adaptive responses. P2YRs respond to nucleotides (ATP, ADP, UTP, UDP) as well as to nucleotide sugars (UDP-glucose) [4–6]. Within the cell, nucleotides and nucleotide sugars occur at high levels and serve as metabolites participating in energy transfer and biosynthetic processes. During homeostasis, extracellular concentrations of these metabolites remain very low. However, during cell and tissue stress, active export or lytic release may cause dramatic increases in extracellular nucleotide (nucleotide sugar) concentrations. Outside the cell, however, these molecules acquire additional functions and become agonists of P2YRs, eliciting signaling cascades with distinct effector responses that may help the cell to cope with stress.

During conditions of stress, which not only include inflammation but also the lack of nutrients (starvation) or oxygen (hypoxia), stressed cells release nucleotides, particularly in the form of ATP or ADP [1]. While these nucleotides can trigger P2YRs, their subsequent degradation by CD39 (ATP → AMP) and CD73 (AMP → adenosine) leads to the accumulation of adenosine and thus to P1 adenosine receptor-mediated cytoprotective effects [7]. In a signaling cascade referred to as the hypoxia-adenosine link, hypoxia increases extracellular adenosine through the induction of CD39 expression and with the participation of hypoxia-inducible factor HIF-1α also the expression of CD73 [8]. The hypoxia-adenosine link can thus serve to prevent cardiac injury myocardial ischemia–reperfusion.

P2YRs have been detected in almost all human tissues. Cell types expressing P2YRs include platelets, T cells, NK cells, dendritic cells, macrophages including microglia (the resident macrophages of the brain parenchyma), neutrophils, endothelial and epithelial cells as well as hepatocytes adipocytes, cardiomyocytes and osteoblasts [9].

P2YR-mediated functions include the induction of cytoskeletal rearrangements, for instance, required during cell shape regulation, cell aggregation and migration as well as endocytosis and phagocytosis. P2YRs also induce and regulate secretory responses [9]. P2YRs may thus exert pro- and anti-inflammatory effects. P2YRs also promote wound healing, angiogenesis and cell survival as well as apoptosis. In the central nervous system (CNS), P2YRs participate in nociception, a neural feedback mechanism that allows the CNS to detect noxious and potentially damaging stimuli in order to initiate protective responses. The expression of several P2YRs in both, osteoblasts and osteoclasts, suggests a role of P2YR signaling in bone biology [10]. P2Y_1_ may regulate osteoclast differentiation and function. P2Y_1_ is most likely osteolytic and may thus promote bone resorption [11]. Likewise, P2Y_6_ supports osteoclast survival as well as osteoclast-mediated bone degradation [12, 13].

The numerous and varied tasks of P2YRs not only in health but also in disease development suggest great therapeutic potential of P2YR targeting. However, clinical development of most P2YRs lags behind. Currently, there are only few examples of P2YR targeting, relating to the treatment of dry eye [14] and thrombotic disease [15].

The ATP receptor P2Y_11_ (encoded by P2RY11) has often been considered an unconventional member of the P2YR family [16, 17]. A major reason why P2Y_11_ enjoys this special reputation is that it couples to G_q_ and to G_s_ proteins [18]. Another distinct feature is its apparent absence in rodents. The resulting lack of P2Y_11_ knockout models has slowed down its exploration. The limited availability of specific antibodies and pharmacological tools has made it even more difficult to study P2Y_11_ expression and function.

Several years ago, we have started to examine P2Y_11_ function using a transcriptomic approach, first in a recombinant cell line and later in human macrophages [19, 20]. A major finding of these studies was P2Y_11_/IL-1 receptor crosstalk. In this review, we discuss how P2Y_11_ cross-communicates with the IL-1 receptor (IL-1R) to control macrophage inflammation and conclude that targeting the P2Y_11_/IL-1R axis might become a promising anti-inflammatory strategy.

## Reprogramming of astrocytoma cells by the ectopic P2Y_11_ receptor

As outlined in previous reviews [16, 17], the limited range of P2Y_11_-selective tools (rodent models, antibodies, agonists, antagonists), has long hampered the efficient examination of its expression and function. For a more systematic approach, we therefore decided to study the P2Y_11_ transcriptome. After several attempts with varying tools, we had to realize that it was difficult if not impossible to establish stable P2Y_11_ transfectants. In retrospect, the best way to explain our failure is that P2Y_11_ activity may not be compatible with productive cell division, which would be in accordance with the earlier observation in endothelial cells that P2Y_11_ impairs cell proliferation by inducing cell cycle arrest [21]. This view is consistent with the fact that the literature provides few significant findings on P2Y_11_ function in cancer cells. Conversely, many meaningful observations have been made in non-proliferating primary cells such as dendritic cells [22–25], macrophages [19, 20, 26–28] and T cells [29–33].

By necessity, we took advantage of a commercial P2Y_11_ recombinant cell line intended for drug discovery and naturally devoid of functional P2 receptors [34, 35]. This glioma cell line originates from 1321N1, a grade II brain astrocytoma, which is homozygous for the TP53 missense mutation (Arg213Gln) in the DNA binding domain of p53 resulting in a drastic reduction of p53 transcriptional activity. The loss of p53 activity prevents activation of cyclin- dependent kinase inhibitor 1a (CDKN1A, encoding p21), a transcriptional target gene of p53 and the central mediator in p53-induced G1 arrest [36, 37]. Due to its inability to induce cell cycle arrest, TP53 Arg213Gln may facilitate stable P2Y_11_ expression without impairing permanent proliferation. Along the same line, coupling of P2Y_11_ to AC in 1321N1 and CHO cell lines turned out to be much weaker than coupling to PLC [38], most likely because cAMP accumulation would not be compatible with effective proliferation [39].

**Fig. 1:**
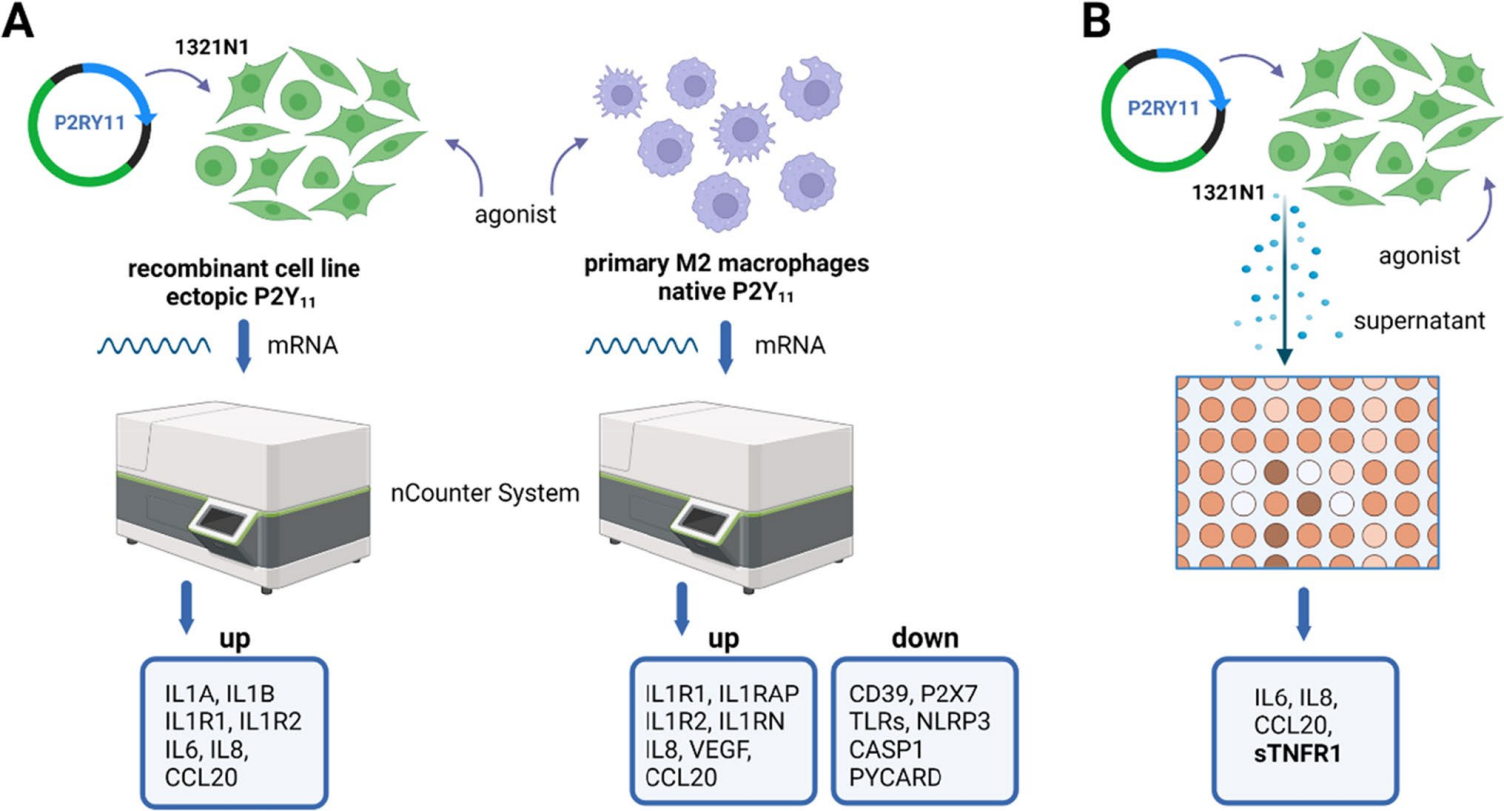
Transcriptomic and secretomic analyses of ectopic and native P2Y_11_ receptors. (**A**) A commercial 1321N1 astrocytoma-based P2RY11-recombinant cell line was used to study ectopic P2Y_11_. A CRISPR/Cas9 knockout cell line served as a negative control in these experiments (not shown). Native P2Y_11_ was examined in human M2 macrophages, which are known to upregulate P2Y_11_ during M-CSF driven differentiation from monocytes. RNA was isolated and subjected to Nanostring analyses. (**B**) Culture supernatants from agonist-treated recombinant cells were examined for the presence of cytokines using an antibody array, which confirmed findings from the Nanostring analyses and additionally identified soluble TNF receptor 1 (sTNFR1) as a P2Y_11_ target

To complete our P2Y_11_ recombinant cell system, we generated an appropriate control cell line using CRISPR/Cas9-mediated gene knockout of the P2RY11 gene [26]. Meis et al*.* not only developed the P2Y_11_ agonist NF546 as well as the P2Y_11_ antagonist NF340, they also demonstrated that IL-8 is a P2Y_11_ target [23]. As a kind of validation of the cell system, we found that P2Y_11_ receptor stimulation triggered the secretion of high levels of IL-8 in the recombinant cell line but not in the knockout control. Using this well-defined experimental system, we performed the first transcriptional profiling of P2Y_11_ activation (Fig. 1A).

We used NanoString technology for our gene expression studies [19, 20, 26]. The NanoString nCounter platform uses unique optical barcodes that hybridize to each target mRNA to enable digital counting of individual RNA molecules without any enzymatic amplification steps. For every mRNA target of interest two adjacent 50 base pair probes complementary to the target region are utilized: a capture probe linked to biotin for immobilization and purification, and a reporter probe connected to a unique colour coded molecular barcode. The gene counts obtained are normalized to the geometric mean of the 40 reference genes. The nCounter CodeSet used was manufactured to additionally contain 8 negative control probes, designed against engineered RNA sequences, which are not present in our biological samples. These negative control probes were used to set background threshold. NanoString technology thus allows quantification of individual mRNAs in side-by-side analyses of cell samples that have or have not been subjected to P2Y_11_ stimulation.

A hallmark of the transcriptional profiles was a strong signature of IL-1 signaling 6 h (but not 24 h) after P2Y_11_ activation [19]. Using both the PanCancer Pathways and the Immunology Panel, we observed P2Y_11_-mediated activation of IL1A, IL1B, IL1R1, IL1RAP and IL1R2. In addition, IL6 and IL8, which are both targets of the IL-1/IL-1R axis, were strongly upregulated. At this point, activation of the genes encoding IL-6 and IL-8 correlated with P2Y_11_-induced secretion of both cytokines observed in the preparatory experiments and thus served as a useful internal control validating the recombinant cell system as well as the NanoString approach. In accordance with P2Y_11_-induced IL-1 signaling, our gene ontology (GO) enrichment analysis revealed that GO terms such as cell surface receptor signaling pathways and cytokine-mediated signaling pathway were clearly over-represented in the set of genes upregulated by P2Y_11_ stimulation.

The strong evidence of cytokine signaling in the transcriptional profile of ectopic P2Y_11_ activation prompted us to continue our studies with a secretome analysis (Fig. 1B). Such an approach not only serves to validate transcriptional changes at the protein level but may also reveal P2Y_11_-driven posttranslational events. A human antibody array kit was used to detect numerous cytokines in the supernatants of P2Y_11_ recombinant 1321N1 astrocytoma cells. In accordance with the mRNA profiles and, consistent with P2Y_11_/IL-1R crosstalk, the IL-1 targets IL-6, IL-8 and CC-chemokine ligand 20 (CCL20; also known as MIP-3α or LARC) accumulated in recombinant 1321N1 cell supernatants with a pattern that clearly demonstrated the specific involvement of P2Y_11_ receptor in the observed response. In accordance with an anti-inflammatory P2Y_11_ function, the soluble form of TNFR1 was released by recombinant 1321N1 cells in response to receptor activation [19]. The release (or shedding) of soluble TNFR1 (sTNFR1) is catalyzed by ADAM17 (A Disintegrin And Metalloprotease 17) also known as TACE [40].

Despite the promising results of the transcriptional and secretomic profiling, significant concerns remained. Are these findings relevant, especially given that p53 is mutated in 1321N1 cells and will therefore fail to execute P2Y_11_-driven programs such as cell cycle arrest? A more physiological system based on non-transformed, primary human cells was clearly required. Additional observations in the NanoString analysis included the upregulation of genes involved in monocyte and macrophage development (CEBPB, NR4A1, PPARG) (unpublished observations). CEBPB encoding C/EBPß not only serves as a transcription factor in IL1, IL6 and IL8 expression but also participates in a cascade that results in the expression of M2 macrophage-specific genes [41]. NR4A1 encoding Nur77 is required for the development of non-classical monocytes and for the differentiation of anti-inflammatory macrophages [42, 43]. PPARG encoding PPAR-γ participates in a signaling pathway that controls differentiation of macrophages from monocytes [44].

In addition, several previous studies of P2Y_11_ had been performed in immune cells and, in particular in myeloid cells, consistent with the view that P2Y_11_ senses extracellular ATP as a damage-associated molecular pattern (DAMP) [45] during stress and immune responses [17, 46]. These considerations collectively prompted us to focus our investigations on macrophages.

## Reprogramming of primary human macrophages by the native P2Y_11_ receptor

Dendritic cells derived from monocytes have previously been used as a cell culture model in P2Y_11_ research [22, 23]. Monocytes also give rise to macrophages [27, 47]. While GM-CSF in combination with IL-4 promotes monocyte differentiation towards dendritic cells, M-CSF drives the conversion of monocytes into macrophages [47]. However, P2Y_11_ expression remains relatively low in dendritic cells. In contrast, P2Y_11_ is strongly upregulated during macrophage differentiation. Consistently, M-CSF stimulates both, P2Y_11_ gene and protein expression [26, 28]. P2Y_11_ surface expression is further increased in the presence of IL-10 [26], which facilitates the development of anti-inflammatory M2c macrophages [48], a macrophage subset that downregulates proinflammatory cytokines, scavenges cell debris and contributes to cell and tissue repair programs.

Just like in dendritic cells, IL-8 also turned out to be a P2Y_11_ target in macrophages [26] and could thus serve as an internal (positive) control. This paved the way for the first transcriptome analysis of the native P2Y_11_ receptor in its natural environment (Fig. 1A). This time [19], we also took the opportunity to consider the role of cAMP signaling by stimulating P2Y_11_ in the presence or absence of rolipram, a phosphodiesterase 4 (PDE4) inhibitor that prevents the rapid breakdown of cAMP [49].

The most important finding up front was the confirmation of the IL-1 signature. Encouragingly, IL1R1 was also strongly upregulated in macrophages by P2Y_11_ activation and enhanced, when rolipram was used to inhibit cAMP degradation [19]. The expression of IL1RAP, which is required for IL-1R function, was also significantly enhanced. This was somewhat surprising because regulation of IL1RAP gene expression had not been described previously [50]. Similar to the recombinant cell line, IL1R2, and in addition IL1RN was also upregulated. The expression of the IL-1 scavenger receptor IL-1R2 or IL-1R antagonist (encoded by IL1RN) could mean, among other things, that the IL-1R response is tightly controlled. In contrast to the recombinant cell line, however, IL1A and IL1B upregulation was not significant in macrophages, suggesting that P2Y_11_-activated macrophages are more prone to respond to exogenous IL-1. IL-1R1 upregulation in response to P2Y_11_ activation was confirmed at the protein level, initially in the recombinant cell line [19, 26] and subsequently in primary human macrophages [19, 20].

The transcriptome analysis in human macrophages also provided further evidence in favor of P2Y_11_ as an anti-inflammatory P2Y receptor. The genes encoding NLRP3 inflammasome components (NLRP3, ASC, CASP1) were all downregulated and expression of several toll-like receptors (TLR5, TLR7, TLR8) was also suppressed [20]. In addition, it provided useful insights into how the receptor's function may be regulated. ATP, the natural P2Y_11_ agonist, is degraded by ecto-enzymes expressed on the cell surface of various cell types. One of the most prominent ATP-hydrolyzing enzymes is the ecto-ATPase CD39 [51], encoded by ENTPD1, which converts ATP via ADP to AMP. P2Y_11_ signaling in human (M2) macrophages appears to be self-sustaining as it delays ATP breakdown by suppressing the expression of CD39, both at the mRNA and the protein level [20]. Prolonged P2Y_11_ signaling through stabilization of its agonist ATP may be critical to the full implementation of the anti-inflammatory program and thus the restoration of homeostasis.

Yet another regulatory mechanism emerged from transcriptional profiling of P2Y_11_ in macrophages. P2Y_11_ induced and rolipram enhanced the expression of suppressor of cytokine signaling 3 (SOCS3) [20], a well established regulator of inflammation [52]. This finding was of particular interest for several reasons. SOCS3 has been reported to control IL-1 signaling by targeting the TRAF6/TAK1 complex [53]. While IL-1R2 and IL-1RA prevent effects of exogenous IL-1, SOCS3 acts intracellulary to control the IL-1 signaling pathway. Moreover, SOCS3 expression is induced by the cAMP effector protein Epac1 (exchange protein directly activated by cAMP 1). Finally, SOCS3 also controls IL-6 signaling [54], explaining our observation that macrophages – in contrast to recombinant 1321N1 cells—fail to produce IL-6 in response to P2Y_11_ activation. Evidence for the functional significance of a cAMP/Epac-1/SOCS3 regulatory axis was obtained through pharmacological Epac-1 inhibition, which enhanced P2Y_11_-driven secretory responses [20].

## The expanding P2Y_11_ secretome

Previous work using the selective antagonist NF340 [23] as well as a CRISPR/Cas9 knockout control had identified IL-6 and IL-8 as bona fide targets of the recombinant P2Y_11_ receptor in the astrocytoma 1321N1 cell line [19]. P2Y_11_-driven IL-8 secretion has been demonstrated in monocyte-derived DCs (moDCs) using the selective agonist NF546 in combination with the selective antagonist NF340 [23]. NF546 activates P2Y_11_, although it belongs to a structural class of antagonists (suramin analogues) [5].

IL-6 production related to P2Y_11_ activation has also been suggested to occur in the human keratinocyte cell line (HaCaT). However, in this cell culture system, P2Y_11_ was not the driving force but instead it enhanced IFNγ-induced IL-6 production [55, 56]. Moreover, P2Y_11_ involvement in the IFNγ-induced IL-6 response was implied by use of NF157, which is however non-selective [23, 57]. 1321N1 astrocytoma cells spontaneously produce IL-8, like many other cancer cell lines [58]. In our hands, NF157 but not NF340 inhibited the spontaneous IL-8 production in 1321N1 cells, which lack P2Y_11_, in a dose-dependent manner (0.5 – 10.0 µM). At 10 µM, NF157-mediated inhibition of IL-8 production was > 70%.

The P2Y_11_ selective agonist ATPγS, a slowly hydrolyzable ATP analog, has also been reported to induce the secretion of IL-6, IL-8, monocyte chemoattractant protein-1 (also known as CCL2), and growth-regulated oncogene α (GROα, also known as CXCL1) [59]. However, due to the use of non-selective antagonists such as pyridoxal-5'-phosphate-6-azophenyl-2',5'-disulfonic acid (PPADS) and suramin, the secretory response induced by ATPγS could not be clearly assigned to P2Y_11_ activity in this study.

VEGF and CCL20 also emerged from our transcriptome analyses. Like IL-6 and IL-8, they are known IL-1R targets [60, 61]. Similar to the recombinant cell system, P2Y_11_ activation in macrophages also resulted in the release of soluble TNF receptors. However, macrophages released sTNFR2 instead of sTNFR1. This is in accordance with the known TNFR expression patterns. While TNFR1 is expressed almost ubiquitously, TNFR2 expression is more restricted to myeloid cells and some other cell types [40]. Moreover, in macrophages but not in astrocytoma cells, the TNFR shedding process could be boosted by PDE inhibition. Importantly, ADAM17 is known to participate in IL-1 signaling cascades [62], again confirming P2Y_11_/IL-1R crosstalk. In addition to the anti-inflammatory effect of TNF-α neutralization, the shedding of sTNFR2 has pro-survival effects as it prevents the pro-cell-death activities of TNFR2 [40].

## IL-1 and cAMP signaling promote P2Y_11_ secretome development

Despite the strong signatures of IL-1 signaling in the recombinant astrocytoma and in human (M2) macrophages, we were unable to detect P2Y_11_-induced IL-1 (IL-1α and IL-1ß) in the supernatants of these cells. The P2Y_11_ targets IL-6 and IL-8 are also considered hallmark cytokines of the senescence-associated secretory phenotype (SASP) [63]. Interestingly, measurement of senescence-associated IL-1 has been difficult, most likely due to the ability of IL-1 to induce substantial responses even at very low concentrations (in the low pg/ml range). While the exact mechanism of P2Y_11_/IL-1R crosstalk currently remains unclear, IL-1R signaling may in principle be induced either by low amounts of IL-1, which are consumed during the stimulatory process, or through an IL-1 independent manner.

**Fig. 2:**
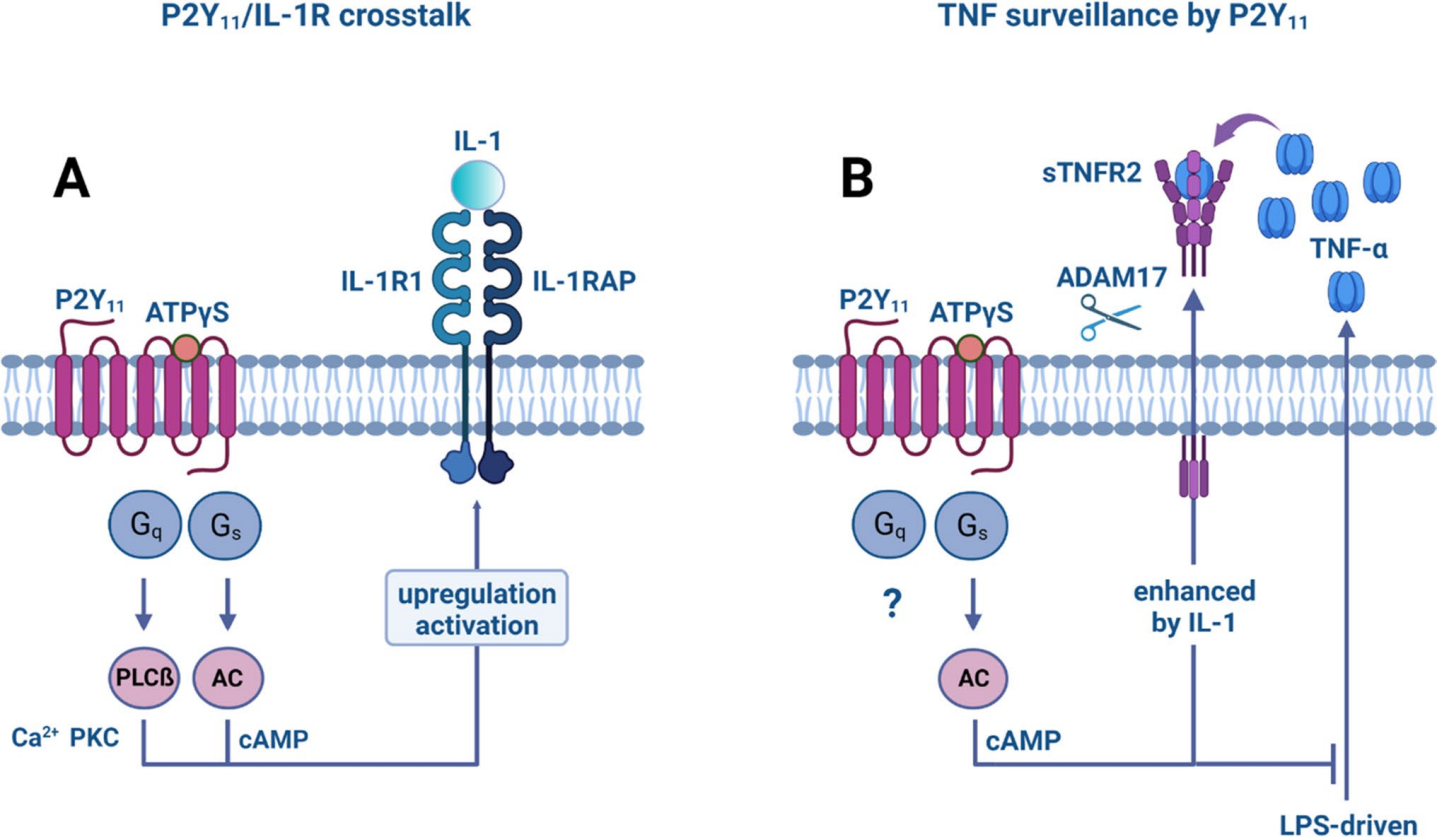
P2Y_11_/IL-1R crosstalk translates into anti-inflammatory responses. (**A**) P2Y_11_ couples to both, G_q_ and G_s_ proteins. While G_q_ activates phospholipase Cß (PLCß) and thus initiates the mobilization of Ca^2+^ (via inositol triphosphate, IP_3_) as well as the activation of PKC (via diacylglycerol, DAG), G_s_ activates adenylyl cyclase (AC) to increase the levels of cyclic AMP (cAMP). All three second messengers of canonical P2Y_11_ signaling participate in the upregulation of IL-1R, thus establishing P2Y_11_/IL-1R crosstalk. (**B**) P2Y_11_ activates two independent mechanisms to control TNF-α driven inflammation. On the one hand, P2Y_11_/IL-1R) activates the sheddase ADAM17/TACE to promote the release of soluble TNF receptors, neutralizing secreted TNF-α. On the other hand, P2Y_11_ effectively suppresses the lipopolysaccharide (LPS)-induced secretion of TNF-α. Both mechanisms engage cyclic AMP signaling to exert these anti-inflammatory effects

Activation of both, ectopic and native P2Y_11_ caused IL-1R upregulation at the mRNA and the protein level [19, 20]. In macrophages, P2Y_11_ mediated IL-1R upregulation was further enhanced by rolipram-induced cAMP accumulation, indicating that one important function of cAMP is to increase cellular sensitivity to low levels of IL-1. In addition to cAMP, calcium and protein kinase C (PKC) were also required for P2Y_11_-driven IL-1R upregulation in macrophages [20]. Obviously, all P2Y_11_ canonical signaling pathways contribute to increased IL-1R expression, thus establishing P2Y_11_/IL-1R crosstalk (Fig. 2A).

This is in line with other studies, demonstrating that cAMP-elevating agents such as prostaglandin E2 (PGE2) [64], phorbol ester mediated PKC activation [65] or calcium ionophore treatment [66] increase IL-1 production. Of note, induction and enhancement of IL-1R expression mediated by phorbol ester and prostaglandin E2 was supported by dexamethasone [65], indicating that IL-1R upregulation can occur in a strongly anti-inflammatory context.

IL-1 signaling is a self-enhancing process because IL-1 not only increases its own expression [67] but also the expression of its receptor [64]. Expression of the potent and potentially pathogenic cytokine IL-1 is controlled by a two-signal mechanism. A first signal, for instance monocyte adherence, may rapidly induce IL-1 and IL-1R mRNA expression [68], however, in the absence of IL-1 and IL-1R protein production. A second signal is necessary to facilitate IL-1 and IL-1R mRNA translation and secretion. In accordance with the self-enhancing principle, IL-1 can act as a second signal and promote its own protein expression [67]. In our macrophage model, the ATP receptor P2Y_11_ may serve as a first (danger) signal, causing the rapid expression of IL-1 and IL-1R mRNAs, which nonetheless remain untranslated or poorly translated. In the presence of IL-1, however, mRNA translation is triggered, allowing the implementation of effective IL-1R signaling.

While the secretome produced by recombinant P2Y_11_ in human astrocytoma cells (1321N1) comprises IL-6 and IL-8 as well as sTNFR1, the secretome generated by the native P2Y_11_ in primary human (M2) macrophages lacks IL-6 but contains the pro-angiogenic factors IL-8 and VEGF as well as sTNFR2 (instead of sTNFR1). Importantly, all secretome components identified either by transcriptomic or secretomic profiling are known to be promoted by IL-1 signaling [19, 20, 26]. Accordingly, reinforcing P2Y_11_/IL-1R crosstalk by the addition of recombinant IL-1α or IL-1ß strongly enhanced all P2Y_11_ secretory responses. At least in macrophages, raising intracellular cAMP levels by use of rolipram further promoted the P2Y_11_/IL-1R driven secretory response, most likely through cAMP-dependent upregulation of IL-1 and IL-1R expression.

## CCL20—a special product of P2Y_11_/IL-1R crosstalk

A P2Y_11_ secretome component that may deserve special attention is the chemokine CCL20, which is outstanding for several reasons. CCL20 (also known as LARC or MIP-3α) emerged as a P2Y_11_ target from the transcriptional profiling of both, the ectopic and the native P2Y_11_ receptor [20]. In fact, CCL20 was among the most strongly activated genes upon ectopic P2Y_11_ stimulation in astrocytoma cells. In macrophages, CCL20 was also strongly activated by P2Y_11_ agonist and even more in the presence of rolipram. Interestingly, only CCL20 was upregulated in response to P2Y_11_ activation among the 24 CCL chemokines analyzed, which implies a strong selectivity in P2Y_11_-induced CCL chemokine activation. Similar to the known two-signal regulation of IL-1 cytokines and IL-1R [67], P2Y_11_-driven CCL20 protein secretion required co-stimulation with recombinant IL-1α or IL-1ß. However, even in the presence of IL-1 cytokines the level of secreted CCL20 protein remained low. Robust P2Y_11_/IL-1R driven CCL20 secretion critically depended on cAMP accumulation induced by rolipram-mediated PDE4 suppression, suggesting that strong IL-1 signaling through an upregulated IL-1R, which is activated by exogenous IL-1 is required for effective CCL20 production and secretion.

CCL20, which is expressed in response to IL-1ß and TNF-α [69], is the ligand of C–C chemokine receptor CCR6 [70]. CCL20 thus recruits DCs as well as T and B cells, which all express CCR6. Both in homeostasis and during inflammation, CCL20 plays a critical role in the skin and at mucosal surfaces. CCL20 may contribute to inflammatory diseases such as psoriasis and ulcerative colitis [71, 72]. Moreover, CCL20 can promote cancer progression through direct effects on cancer cells and indirectly by remodeling the tumor microenvironment [73]. CCL20 serum levels have been proposed as a useful biomarker for for the early differential diagnosis between benign tumors and ovarian cancer [74]. By regulating the migration of regulatory T cells (Tregs) [75], CCL20 may differentially affect inflammatory processes and tumorigenesis [76]. CCL20 has also been implicated in the recruitment of IL17 producing helper T (Th17) cells, which exhibit pro-inflammatory as well as pro-angiogenic effects but may also be central to tissue repair and regeneration [77].

The CCL20/CCR6 axis also participates in hepatic angiogenesis [78]. CCL20 has been shown to be a direct pro-angiogenic factor that induces endothelial cell invasion, sprouting and migration through acting on endothelial CCR6.

It is currently unclear whether P2Y_11_-induced CCL20 is pro- or anti-inflammatory. In our experimental system, CCL20 is produced in the absence of TNF-α. Moreover, increased levels of cAMP are critically required for effective CCL20 generation and secretion, altogether suggesting that P2Y_11_-induced CCL20 more likely serves anti-inflammatory and homeostatic purposes.

## P2Y_11_ acts as a sentinel of TNF-α induced inflammation

Transcriptional and secretomic profiling revealed numerous clues for P2Y_11_ as an anti-inflammatory receptor. However, the most impressive anti-inflammatory effects of P2Y_11_ related to the surveillance of TNF-α induced inflammation. P2Y_11_ is obviously capable of controlling TNF-α at two different levels [19, 20] (Fig. 2B). On the one hand, P2Y_11_ induces the ADAM17-dependent release of soluble TNF receptors to neutralize pre-existing TNF-α, and on the other, P2Y_11_ suppresses the LPS-driven de novo biosynthesis of TNF-α. Both mechanisms depend on or are enhanced by cyclic AMP signaling. Collectively, these observations clearly implicate P2Y_11_ receptors in the surveillance of TNF-α.

TNF-neutralizing biopharmaceuticals are among the most successful drugs for the treatment of inflammatory and autoimmune diseases [79]. TNF-α not only induces inflammation, it may also cause cell death, when distinct checkpoints are inactivated. Although TNF-induced cell death may be desirable during pathogen defense, it may aggravate inflammatory processes and contribute to pathogenesis. The combination of TNF-induced inflammation and cell death may be responsible for the cytokine storm, known to occur in life-threatening conditions such as sepsis and severe COVID-19. Accordingly, neutralizing antibodies to TNF (and IFNγ) have been shown to protect mice from death during SARS-CoV-2 infection [80]. Thus, the ability of P2Y_11_ to promote TNF neutralization via induction of TNFR shedding and to suppress TLR-driven TNF production through cyclic AMP signaling recommend P2Y_11_ as a target of anti-inflammatory strategies.

## Conclusions

P2Y_11_ is the only P2Y family member coupling to both, PLCß and AC, via G_q_ and G_s_, respectively [18]. However, in recombinant cell systems used in the past and based on cancer cell lines, coupling to AC via Gs appeared to be much weaker leading to an underestimation of cAMP signaling in P2Y_11_ responses. In contrast to ectopic P2Y_11_ receptors, virtually all responses driven by the native P2Y_11_ in macrophages could be strongly enhanced by rolipram, which inhibits PDE4, a major PDE isoform in macrophages [19, 20]. These observations emphasize the role of the G_s_-AC axis in native P2Y_11_ receptor signaling and suggest that P2Y_11_ receptor signaling and function should preferentially be studied in primary (immune) cells.

Our recent studies indicate that P2Y_11_ engages IL-1R signaling but the exact mechanism is still unclear and requires further examination. Although increasing observations argue in favor of P2Y_11_ as an anti-inflammatory P2YR, for instance by controlling TNF-α, it cannot be excluded that P2Y_11_ also participates in pro-inflammatory responses, for instance by inducing CCL20 production. The concept of P2Y_11_ as an adaptive receptor that stimulates or inhibits inflammation, cell migration and possibly also cell metabolism [81] in a context-dependent manner still deserves further evaluation. If P2Y_11_ indeed turns out to be mainly anti-inflammatory and P2Y_11_/IL-1R crosstalk emerges as a requirement for adaptive and homeostatic responses, it might become relevant in the clinical concept of IL-1R blockade for the treatment of inflammatory diseases [82].

## Data Availability

Not applicable.
